# Methamphetamine neurotoxicity, microglia, and neuroinflammation

**DOI:** 10.1186/s12974-018-1385-0

**Published:** 2018-12-12

**Authors:** Fatemeh Shaerzadeh, Wolfgang J. Streit, Soomaayeh Heysieattalab, Habibeh Khoshbouei

**Affiliations:** 1 Department of Neuroscience, University of Florida College of Medicine and McKnight Brain Institute, JHM Health Science Center, PO Box 100244, Gainesville, FL 32610 USA; 2 Cognitive Neuroscience Division, Faculty of Education and Psychology, University of Tabriz, Tabriz, Iran

**Keywords:** Microglia, Methamphetamine, Dopamine, Neuroimmune

## Abstract

Methamphetamine (METH) is an illicit psychostimulant that is subject to abuse worldwide. While the modulatory effects of METH on dopamine neurotransmission and its neurotoxicity in the central nervous system are well studied, METH’s effects on modulating microglial neuroimmune functions and on eliciting neuroinflammation to affect dopaminergic neurotoxicity has attracted considerable attention in recent years. The current review illuminates METH-induced neurotoxicity from a neuropathological perspective by summarizing studies reporting microglial activation after METH administration in rodents. Assessing microglial reactivity in terms of the cells’ morphology and immunophenotype offers an opportunity for comprehensive and objective assessment of the severity and nature of METH-induced neuronal perturbations in the CNS and can thus contribute to a better understanding of the nature of METH toxicity. We reach the conclusion here that the intensity of microglial activation reported in the majority of animal models after METH administration is quite modest, indicating that the extent of dopaminergic neuron damage directly caused by this neurotoxicant is relatively minor. Our conclusion stands in contrast to claims of excessive and detrimental neuroinflammation believed to contribute and exacerbate METH neurotoxicity. Thus, our analysis of published studies does not support the idea that suppression of microglial activity with anti-inflammatory agents could yield beneficial effects in terms of treating addiction disorders.

## Background

The effects of METH in the central nervous system (CNS) are well studied, and for some time now, METH has been reported to increase dopamine (DA) neurotransmission through regulation of dopamine transporter activity [1–3], and to cause neurotoxic effects, notably the apparent degeneration of dopaminergic terminals in the striatum [4]. The responses of microglia, as well as those of astrocytes, have been studied following METH administration to laboratory animals, and our objective here is to summarize and critically evaluate methamphetamine-induced, histopathologically evident microglial responses that occur following METH administration to rodents. Neuroimmune activity involving microglia has been implicated in METH toxicity in the sense that microglial activation is thought to contribute to neurotoxicity [5, 6], but this role of microglia remains mostly speculative for a variety of reasons discussed in the paragraphs following.

We view microglia as “sensors of pathology,” cells that continuously monitor neuronal well-being and become alerted (activated) when neuronal activity is disturbed or compromised by injury, disease, or neurotoxic agents. Microglia are thus the first line of defense against toxic insults and most sensitive sentinels of neurotoxic effects and neuronal damage in general. Their responses to disturbed neuronal homeostasis likely result in neuroprotective and/or reparative mechanisms in order to minimize neuronal damage. As we discuss here, the experimental evidence available does indeed support this notion by showing relatively mild activation of microglia after METH and little, if any, evidence of frank degeneration of DA neurons.

## METH neurotoxicity and reactive gliosis

**Fig. 1:**
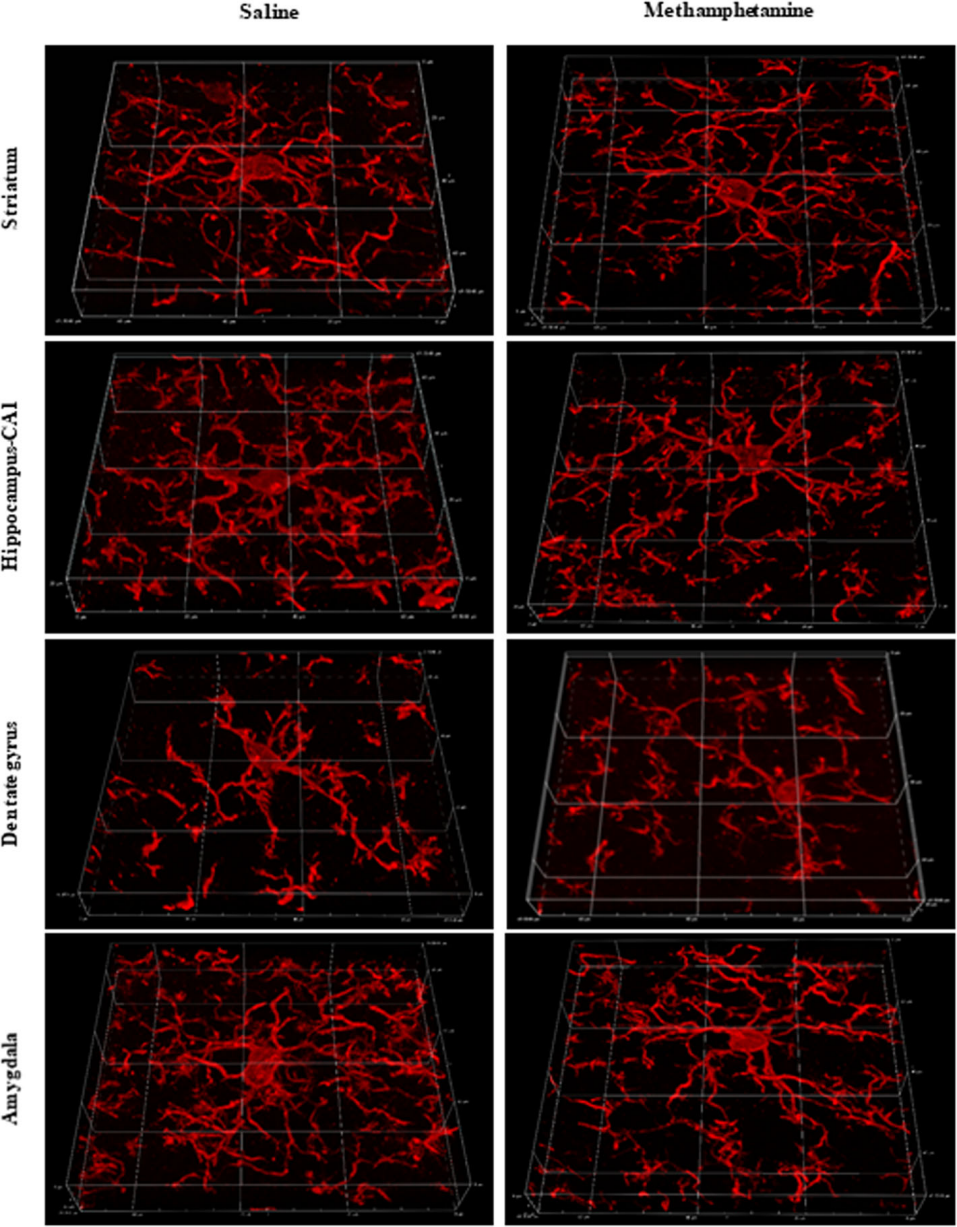
Immunohistochemical staining of Iba1 antigen for visualizing mouse microglial cells after METH administration. 3D reconstructions of 60× stacked images of microglia from striatum, CA1 and dentate gyrus of hippocampus, and from amygdala after 7 days of single, daily i.p. injections of either saline or METH (4 mg/kg). Note the absence of microglial hypertrophy (activation) after METH suggesting minimal neurotoxic damage in this particular injection paradigm. Graticule size, 20 μm

For a variety of reasons methamphetamine’s neurotoxic effects, although well-studied, are not well-defined [6]. In experimental studies, variations of METH doses, routes of administration, duration of METH exposure, and species specificity have collectively confounded the interpretation of the neurotoxic effects of METH and therefore their pathophysiological significance for humans (Fig. 1). Multiple mechanisms are thought to mediate METH-induced neurotoxicity: increase in neuronal firing rate, increased concentrations of intracellular Ca^+ 2^ and Na^+^ ions, dysregulation of mitochondrial function, neuronal energetic imbalance, and overproduction of reactive oxygen species. The primary goal of the current review is to re-evaluate this neurotoxicity from the perspective of reactive microglial cell changes, as neuroinflammatory reactions have been reported to occur following METH administration and are believed to causally contribute to METH-induced neurotoxicity [5–7].

Rather than viewing microglia as potentially harmful cells that attack neurons, we view microglia as sensitive biological indicators of neuronal perturbations and perhaps the most reliable cellular sentinels of neurotoxic effects and neuronal damage in general. Microglia have long been recognized as “sensors of pathology” [8] because they are continuously monitoring neuronal well-being and become alerted (activated) when neuronal activity is disturbed or compromised by injury, disease, or neurotoxic agents. There is a regular and bidirectional crosstalk between microglia and dopamine neurons involving diverse receptors and ligands, including both D1-like and D2-like dopamine receptors [9]. Therefore, increased or decreased extracellular dopamine levels are predicted to affect the biology of microglial cells and dopamine neurons in a bidirectional manner. As the brain’s immune system, one of the prime functions of microglia is to be the first line of defense and engage in neuroprotection whenever necessary [10, 11]. To this end, microglia need to sense which neuronal perturbations require their attention and intervention.

We understand the term “neurotoxicity” to mean that METH exposure is directly toxic to neurons, but not necessarily in the sense that METH kills neurons. Dopaminergic neurons are of particular interest because METH is known to directly target dopamine neurons; it competes with the uptake of released dopamine via the dopamine transporter (DAT); it disrupts vesicular storage of dopamine; and induces reverse transport of dopamine through the transporter termed “dopamine efflux” [12–15]. These actions of METH result in extracellular dopamine concentrations remaining elevated while METH is present, but it is still being debated whether or not elevated dopamine itself is neurotoxic. Neurotoxicity is indicated by the sequelae of METH exposure, which include degeneration of axon terminals in the striatum, as shown by silver cupric impregnation [16, 17], neurochemical changes, such as decreased tyrosine hydroxylase (TH) and DAT levels, depletion of dopamine and its metabolites [16, 18–20], as well as reactive astrogliosis [16] and microgliosis (next paragraph). Reactive astrogliosis (astrocytic scarring) typically follows an initial response by microglial cells. Robust and persistent increases in the astroglial intermediate filament protein, GFAP, have been measured [21], but increased GFAP levels allow no more than a general conclusion that sustained astroglial scarring has occurred due to some (unspecified) neuronal damage. Measurements of GFAP expression do not allow conclusions about whether or not neuronal death has occurred acutely. Neuronal death is the most severe consequence of neurotoxicity and the primary cause for neuronal loss.

Microglial reactions visualized histopathologically are more telling than biochemical measures of GFAP in that the microscopic observation of microglial responses better inform about the regional specificity, nature, and severity of METH-induced neurotoxicity. Not only do microglia display different morphological forms after a damaging event, their surface immunophenotype is marked by presence of multiple membrane-bound receptors that undergo characteristic changes during activation, which are detectable with antibodies directed against a variety of immunological important molecules, such as major histocompatibility (MHC) antigens, complement and Fc receptors, integrins, surface immunoglobulins, and toll-like receptors, in addition to presence of intracellular microglial antigens, like the ionized calcium-binding adapter molecule 1 recognized by the Iba1 antibody [22]. Nevertheless, these immunophenotypical and microanatomical changes are neither global nor universal amongst different species and can vary substantially in accordance with lesion severity thus allowing refined assessments of pathology.

## Regional sensitivity and species specificity of METH modulation of microglial activity

In mice exposed to METH, there are noticeable differences in the nature and intensity of microglial activation that occurs in the nigrostriatal dopaminergic system versus how microglia respond in limbic structures, such as hippocampus and amygdala. While the effects of METH on microglial biology in the ventral tegmental area (VTA) are unclear and there are no reports of microglial activation occurring around VTA dopaminergic neurons, in the striatum where dopaminergic terminal degeneration has been suggested by positive silver staining and decreased levels of dopamine and TH [16, 23, 24], microglia show only mild hypertrophy (hyperramification). Microglial hypertrophy is *the* critical morphological feature for identifying activated microglia. Studies show that in the striatum microglia remain ramified, but upregulate isolectin B4 binding relative to controls where microglia are unstained [25–27]. In addition, the loss of TH immunoreactivity in the striatum appears to be reversible, is affected by hyperthermia, and its association with neurodegeneration is asymmetrical in that Fluoro-Jade C staining is reported in one hemisphere only [24]. The situation is similar in the substantia nigra where a rather mild increase in Mac-1 immunoreactivity has been shown to occur on ramified microglia [23] but no upregulation of isolectin B4 binding [25]. These observations regarding microglia in the dopaminergic system point towards minimal damage of DA neurons following METH exposure. They stand in stark contrast to what is described in the hippocampus and amygdala where microglial hypertrophy is pronounced and indicative of phagocytosis [28]. Together with a positive signal for Fluoro-Jade C indicating neurodegeneration, it is likely that significant neuronal death occurs in these limbic structures. In contrast, METH exposure in rats reveals little or no evidence for neuronal damage in the hippocampus, but instead prominent neurodegeneration in the thalamus in parallel with robust microglial activation [29]. Interestingly, many activated microglia in the thalamus and parietal cortex are seen in close association with the vasculature pointing towards methamphetamine-induced vascular damage and BBB compromise. Both vascular toxicity and neurotoxicity are exacerbated by high blood corticosterone pretreatment [30].

## Vascular toxicity of METH and its effects on reactive microgliosis

The direct effects of METH are not limited to neurons; there is also vascular toxicity and blood-brain barrier (BBB) damage, as well as hyperthermia and seizures that contribute to neurotoxicity and to microglial activation. Not surprisingly, hyperthermia and neuroinflammation are much exacerbated if animals receive bacterial lipopolysaccharide after METH [31]. While there are differences between mice and rats [32], generally METH-induced hyperthermia appears to exacerbate neurotoxicity via disruption of ion channel functions and reactive oxygen species overproduction, as well as through vascular leakage [33]. It has been shown that BBB breakdown occurs in the septum, hippocampus, and amygdala of mice and rats after exposure to acute, very high doses of METH [28, 32]. The high-dose regimen of METH can disrupt integrity and function of the BBB particularly when accompanied by hyperthermia and increased brain temperature. Focal areas of vascular leakage and BBB permeability have been reported in specific brain regions in hyperthermic rats when body temperatures are ≥ 41.7 °C [34]. Bowyer and colleagues showed that following METH injection, activated microglia dramatically increased around the vasculature with or without minimal neurodegeneration. There was a positive correlation between number of activated microglia in septum, hippocampus, and intralaminar, ventromedial, and ventrolateral thalamus nuclei with the number of the episodes of peak body temperatures ≥ 41.7 °C in animals sacrificed 3 days after METH exposure [29]. It appears that activation of microglia surrounding regions of vascular damage is influenced by microglia interacting with blood-borne factors leaking into the CNS rather than as a direct impact of METH on microglia. These results suggest that microglia are not involved in either the initiation or the progression of METH-induced neurodegeneration. Instead this is more likely to occur as a consequence of direct METH-induced vascular toxicity.

## Effects of METH on the immune system

METH is reported to affect immune function in multiple and generally suppressive ways, which were reviewed recently [6]. In terms of how METH toxicity may directly affect microglia, there has been much speculation about excessive microglial activation contributing to or exacerbating brain damage, but it is not clear what evidence there is to support this idea, e.g., at what point does microglial activation become excessive? Proinflammatory cytokines and chemokines are frequently cited as mediators of brain damage, but these substances are produced all the time in the brain, where they serve as intercellular signaling molecules as well as potential modulators of classical neurotransmission [35]. The idea of microglia producing neurotoxins and proinflammatory cytokines that exacerbate brain damage has had a longstanding history and has been implicated in other scenarios, such as CNS trauma and neurodegenerative diseases despite the lack of compelling in vivo evidence showing microglia-mediated neurodegeneration. The notion that activated microglia are harmful is rooted primarily in cell culture studies [11].

The claim that METH-induced neuroinflammation perpetuates neurodegeneration is speculative and unproven, and it is unclear whether it contributes to METH-induced neurotoxicity [36]. We are not promoting this hypothesis because it has not been shown that microglial activation causes neurodegeneration or is toxic in vivo. Rather, microglial activation is a consequence of neurotoxicity exerted by METH and other agents; it represents the cellular response to neuronal damage and is most likely aimed at restoring homeostasis. From the discussion in the paragraphs above, it seems clear that microglial activation observed after METH is the result of both neuronal and vascular injury induced by METH. Recent work has identified a potential mechanism by which damaged neurons release danger-associated molecular patterns, such as high-mobility group box-1 (HMGB1), and/or through purinoceptors (P2X7R) that could mediate signaling between neurons damaged by METH and surrounding microglial cells [37, 38]. There is also pharmacological evidence to support a lack of involvement of neuroinflammation in METH neurotoxicity, for example, lowering core temperature with ethanol can block neuroinflammation, which means that it is the tissue damage that initiates neuroinflammation, not the other way around [39].

Additional evidence against the idea that neuroinflammation causes neurodegeneration stem from observations that the microglial response in the striatum after METH administration is quite mild and can be characterized as minimal microglial activation, if it occurs at all (Fig. 1). This slight microglial activation is unlikely to be causing degeneration of axon terminals in the striatum. In fact, electron microscopy studies, which represent the gold standard for unequivocally demonstrating degeneration, have produced no evidence of axon terminal degeneration in this region [40]. We agree with these authors that the lack of ultrastructural evidence together with rapid recovery of TH immunoreactivity in the striatum is indicative merely of disturbed dopamine biosynthesis rather than some form of severe or even lethal neurotoxic injury. Moreover, the microgliosis that occurs appears acutely after binge-dosed METH administration but then becomes attenuated over time in parallel with recovery of TH immunoreactivity while astroglial scarring persists [21, 36], which is typical for other neurotoxicants that produce reactive gliosis [41]. Microglial activation also decreases rapidly after chronic METH exposure suggesting dissipation of the acute neurotoxic insult [42].

## Conclusions

**Fig. 2:**
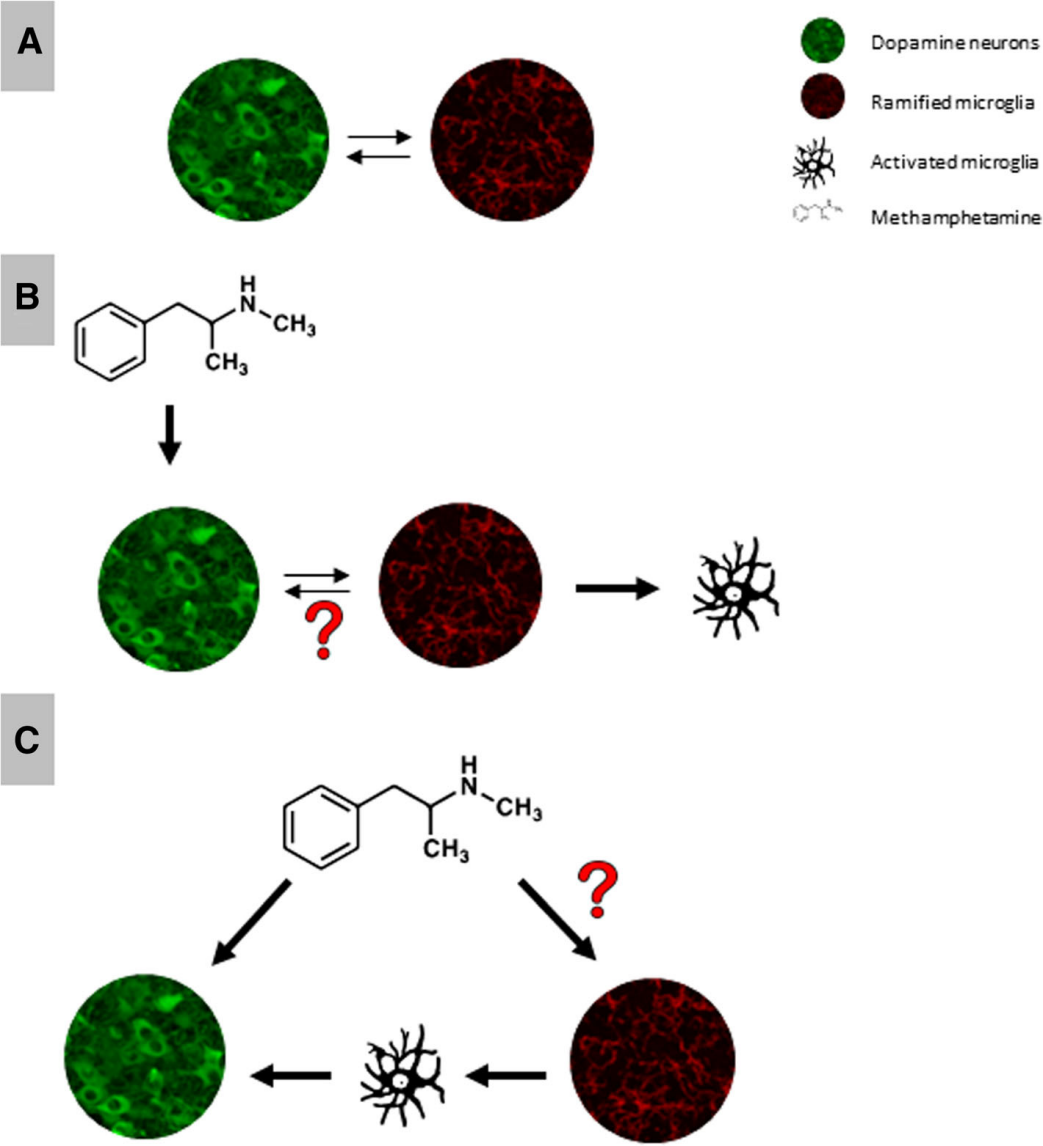
**a** Under normal condition (absence of METH), there are reciprocal interactions occurring between DA neurons and microglia. **b** METH-mediated dysregulation of DA neurons alters neuronal-microglial interactions leading to mild microglial activation; however, the intercellular signaling is unknown. **c** It is unknown whether or not METH can directly induce microglial activation or otherwise alter microglial function to influence its neurotoxicity

In summary, immune responses are not required to elicit METH neurotoxicity, rather the immune reaction is a response to the damaging effects of this toxic substance on neurons. Whether or not microglia play an important role in cellular mechanisms of addiction remains an interesting question at this time that will require further investigation (Fig. 2).

## Abbreviations

BBB: Blood-brain barrier
CNS: Central nervous system
DA: Dopamine
DAT: Dopamine transporter
GFAP: Glial fibrillary acidic protein
METH: Methamphetamine
TH: Tyrosine hydroxylase
VTA: Ventral tegmental area
